# Crystal structure of 2-{[(5-nitro­thio­phen-2-yl)methyl­idene]amino}­phenol

**DOI:** 10.1107/S2056989015009202

**Published:** 2015-05-23

**Authors:** Figen Koçak, Hasan Tanak, Erbil Ağar, Onur Erman Doğan, Namık Özdemir

**Affiliations:** a Department of Physics, Faculty of Arts & Science, Amasya University, TR-05100 Amasya, Turkey; b Department of Chemistry, Faculty of Arts & Science, Ondokuz Mayıs University, 55139 Samsun, Turkey; c Department of Chemistry, Faculty of Arts & Science, Ondokuz Mayıs University, TR-55139 Kurupelit-Samsun, Turkey; d Department of Physics, Ondokuz Mayıs University, TR-55139 Samsun, Turkey

**Keywords:** crystal structure, Schiff bases, phenol, hydrogen bonding, π–π stacking

## Abstract

The title compound, C_11_H_8_N_2_O_3_S, is roughly planar; the di­hedral angle between the planes of the thio­phene and benzene rings is 8.38 (10)°. An intra­molecular O—H⋯N hydrogen bond generates an *S*(5) ring motif. In the crystal, mol­ecules are linked into centrosymmetric dimers by pairs of O—H⋯O hydrogen bonds with an *R*_2_^2^(22) graph-set motif. Aromatic π–π stacking inter­actions [centroid–centroid sep­ar­ations = 3.653 (3) and 3.852 (3) Å] link the dimers into a three-dimensional network.

## Related literature   

For Schiff bases as ligands, see: Aydoğan *et al.* (2001 ▸); Tanak *et al.* (2009 ▸). For related structures, see: Tanak *et al.* (2013 ▸, 2014 ▸). 
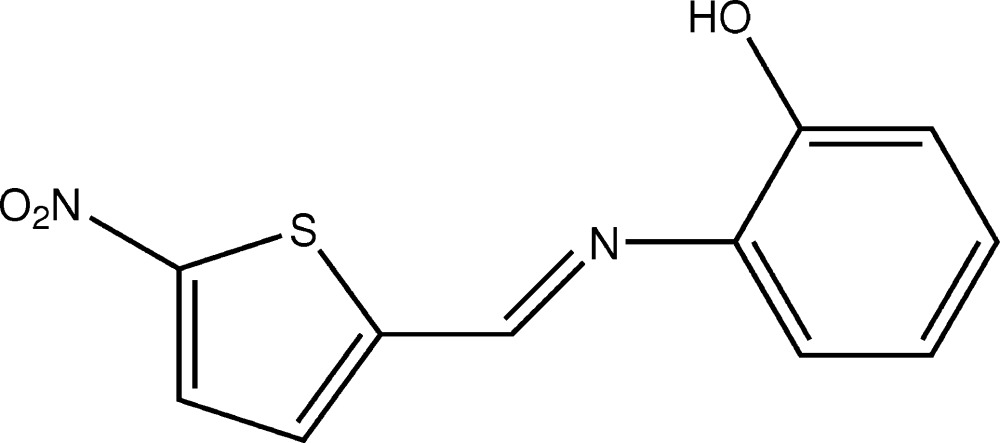


## Experimental   

### Crystal data  

C_11_H_8_N_2_O_3_S
*M* *_r_* = 248.25
Monoclinic, 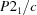

*a* = 10.642 (5) Å
*b* = 7.043 (5) Å
*c* = 14.535 (5) Å
β = 93.566 (5)°
*V* = 1087.3 (10) Å^3^
*Z* = 4
Mo *K*α radiation
μ = 0.29 mm^−1^
*T* = 293 K
0.68 × 0.37 × 0.15 mm

### Data collection  

Stoe IPDS diffractometer
Absorption correction: integration (*X-RED32*; Stoe & Cie, 2002 ▸) *T*_min_ = 0.877, *T*_max_ = 0.965
7883 measured reflections
2254 independent reflections
1696 reflections with *I* > 2σ(*I*)
*R*_int_ = 0.114

### Refinement  

*R*[*F* ^2^ > 2σ(*F* ^2^)] = 0.064
*wR*(*F* ^2^) = 0.100
*S* = 0.98
2254 reflections
186 parameters
H atoms treated by a mixture of independent and constrained refinement
Δρ_max_ = 0.89 e Å^−3^
Δρ_min_ = −0.45 e Å^−3^

### 

Data collection: *X-AREA* (Stoe & Cie, 2002 ▸); cell refinement: *X-AREA* ; data reduction: *X-RED32* (Stoe & Cie, 2002 ▸); program(s) used to solve structure: *SHELXS97* (Sheldrick, 2008 ▸); program(s) used to refine structure: *SHELXL97* (Sheldrick, 2008 ▸); molecular graphics: *ORTEP-3 for Windows* (Farrugia, 2012 ▸); software used to prepare material for publication: *WinGX* (Farrugia, 2012 ▸).

## Supplementary Material

Crystal structure: contains datablock(s) I. DOI: 10.1107/S2056989015009202/hb7421sup1.cif

Structure factors: contains datablock(s) I. DOI: 10.1107/S2056989015009202/hb7421Isup2.hkl

Supporting information file. DOI: 10.1107/S2056989015009202/hb7421Isup3.cml

**Fig. 1.**
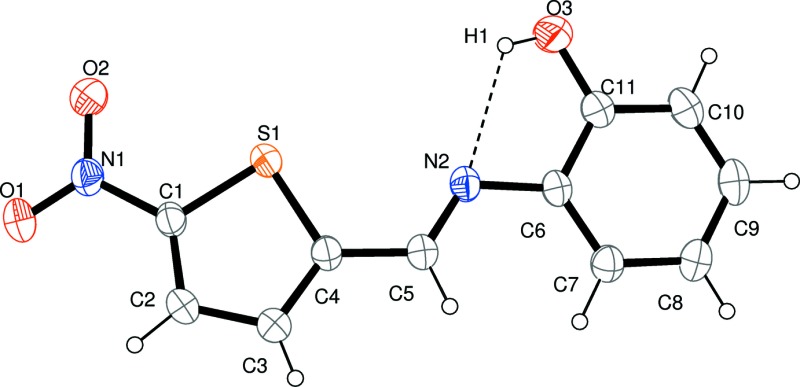
The molecular structure of the title compound, showing 30% probability diplacement ellipsoids.

. DOI: 10.1107/S2056989015009202/hb7421fig1.tif
The mol­ecular structure of the title compound, showing 30% probability diplacement ellipsoids.

**Fig. 2.**
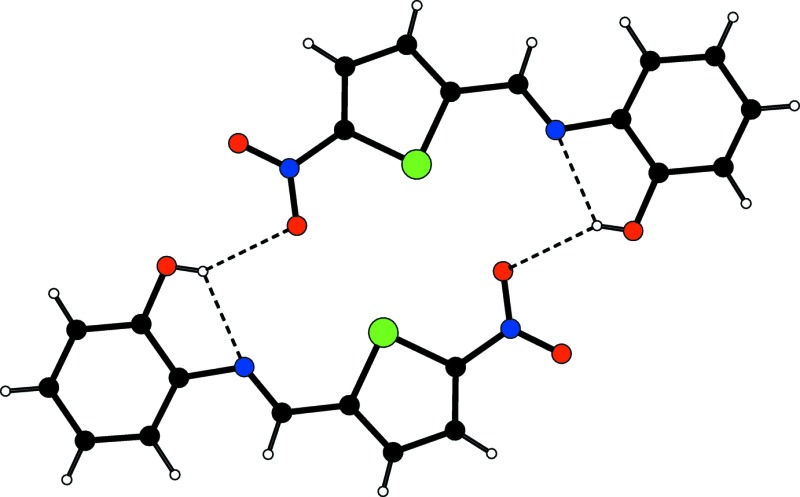
Centrosymmetric dimer with a central R22(22) ring motif. Dashed lines indicate hydrogen bonds.

. DOI: 10.1107/S2056989015009202/hb7421fig2.tif
Centrosymmetric dimer with a central 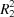
(22) ring motif. Dashed lines indicate hydrogen bonds.

**Fig. 3.**
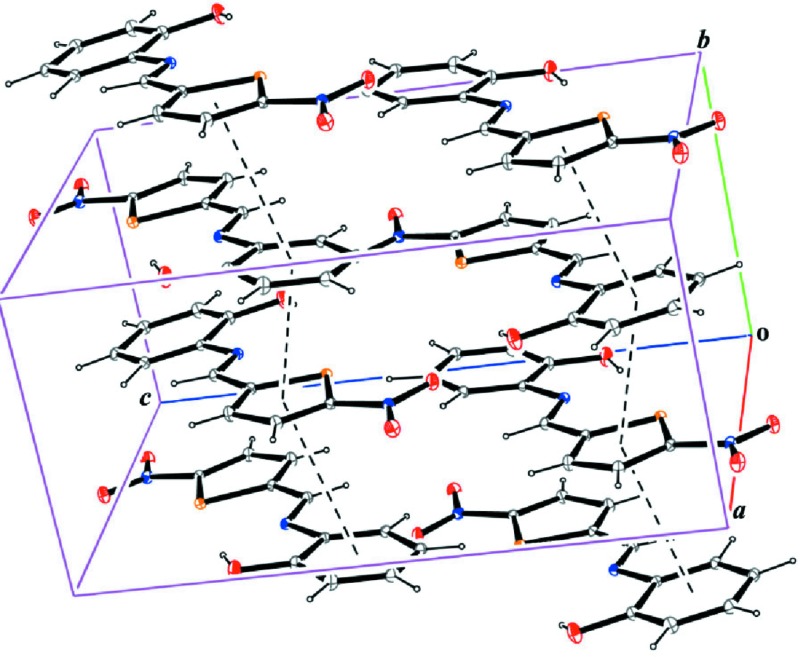
Packing diagram of the title compound.

. DOI: 10.1107/S2056989015009202/hb7421fig3.tif
Packing diagram of the title compound.

CCDC reference: 1400935

Additional supporting information:  crystallographic information; 3D view; checkCIF report

## Figures and Tables

**Table 1:** Hydrogen-bond geometry (, )

*D*H*A*	*D*H	H*A*	*D* *A*	*D*H*A*
O3H1O2^i^	0.78(3)	2.54(3)	3.192(3)	141(3)
O3H1N2	0.78(3)	2.23(3)	2.711(2)	121(3)

Symmetry code: (i) 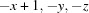.

## supplementary crystallographic information

### S1. Comment

Schiff bases have long been employed as ligands for the complexation of metal ions (Aydoğan *et al.*, 2001; Tanak *et al.*, 2009).

In the title compound (Fig. 1), the molecular structure is almost planar. The dihedral angle between the C1—C4/S1 thiophene and the C6—C11 phenyl rings is 8.38 (10)°. The imino group is coplanar with the nitrothiophene ring as it can be shown by the C3–C4–C5–N2 torsion angle is 178.60 (19)°. The length of the C5=N2 double bond is 1.264 (2) Å, it is slightly shorter than standart 1.28 Å value of C=N double bond and consistent with the related stuructures (Tanak *et al.*, 2013; Tanak *et al.*, 2014). The C1–S1 and C4–S1 bond lengths of the thiophene ring are slightly different than the accepted value for an C*sp*^2^–S single bond (1.76 Å), resulting from the conjugation of the electrons of atom S1 with atoms C1 and C4 (Tanak *et al.*, 2014).

The crystal structure is stabilized by O–H···N and O–H···O type intra and intermolecular hydrogen bonds. An intramolecular O3—H1···N2 interaction (Table 1 and Fig. 1) generates an S(5) ring motif, 1995). In the crystal structure, pairs of O3—H1···O2 hydrogen bond link the molecules to form inversion dimer (Fig. 2) with an *R*_2_^2^(22) ring motif. The crystal structure also feaures π–π stacking interactions with distances of *Cg*1···*Cg*2 = 3.653 (3) Å [symmetry code = 1 - *x*,-1/2 + *y*,1/2 - *z*] and *Cg*1···*Cg*2 = 3.852 (3) Å [symmetry code = 1 - *x*,1/2 + *y*,1/2 - *z*], where *Cg*1 and *Cg*2 are the centroids of C1—C4/S1 and C6—C11 rings, respectively. The details of the hydrogen bonds are summarized in Table 1. A packing diagram of the title compound is shown in Fig. 3.

### S2. Experimental

The title compound was prepared by refluxing a mixture of a solution containing 5-nitrothiophene-2-carbaldehyde (18.4 mg, 0.117 mmol) in ethanol (20 ml) and a solution containing 2-aminophenol (12.8 mg, 0.117 mmol) in ethanol (20 ml). The reaction mixture was stirred for 5 h under reflux. Single crystals of the title compound for X-ray analysis were obtained by slow evaporation of an ethanol solution (yield 60%; m.p. 430–432 K).

### S3. Refinement

C-bound H atoms were positioned geometrically and refined using a riding model, with C—H = 0.93 Å and *U*_iso_(H) = 1.2*U*_eq_(C). The position of the H1 atom was obtained from a difference map of the electron density in the unit-cell and was refined freely.

### Crystal data

**Table:**

C_11_H_8_N_2_O_3_S	*F*(000) = 512
*M**_r_* = 248.25	*D*_x_ = 1.516 Mg m^−^^3^
Monoclinic, *P*2_1_/*c*	Mo *K*α radiation, λ = 0.71069 Å
Hall symbol: -P 2ybc	Cell parameters from 9881 reflections
*a* = 10.642 (5) Å	θ = 1.9–29.0°
*b* = 7.043 (5) Å	µ = 0.29 mm^−^^1^
*c* = 14.535 (5) Å	*T* = 293 K
β = 93.566 (5)°	Prism, dark brown
*V* = 1087.3 (10) Å^3^	0.68 × 0.37 × 0.15 mm
*Z* = 4	

### Data collection

**Table:**

Stoe IPDS diffractometer	2254 independent reflections
Radiation source: fine-focus sealed tube	1696 reflections with *I* > 2σ(*I*)
Graphite monochromator	*R*_int_ = 0.114
Detector resolution: 6.67 pixels mm^-1^	θ_max_ = 26.5°, θ_min_ = 1.9°
rotation method scans	*h* = −13→13
Absorption correction: integration (*X-RED32*; Stoe & Cie, 2002)	*k* = −8→8
*T*_min_ = 0.877, *T*_max_ = 0.965	*l* = −18→18
7883 measured reflections	

### Refinement

**Table:**

Refinement on *F*^2^	Primary atom site location: structure-invariant direct methods
Least-squares matrix: full	Secondary atom site location: difference Fourier map
*R*[*F*^2^ > 2σ(*F*^2^)] = 0.064	Hydrogen site location: inferred from neighbouring sites
*wR*(*F*^2^) = 0.100	H atoms treated by a mixture of independent and constrained refinement
*S* = 0.98	*w* = 1/[σ^2^(*F*_o_^2^) + (0.0675*P*)^2^] where *P* = (*F*_o_^2^ + 2*F*_c_^2^)/3
2254 reflections	(Δ/σ)_max_ < 0.001
186 parameters	Δρ_max_ = 0.89 e Å^−^^3^
0 restraints	Δρ_min_ = −0.45 e Å^−^^3^

### Special details

**Table:**

Experimental. 360 frames, detector distance = 80 mm
Geometry. All e.s.d.'s (except the e.s.d. in the dihedral angle between two l.s. planes) are estimated using the full covariance matrix. The cell e.s.d.'s are taken into account individually in the estimation of e.s.d.'s in distances, angles and torsion angles; correlations between e.s.d.'s in cell parameters are only used when they are defined by crystal symmetry. An approximate (isotropic) treatment of cell e.s.d.'s is used for estimating e.s.d.'s involving l.s. planes.
Refinement. Refinement of *F*^2^ against ALL reflections. The weighted *R*-factor *wR* and goodness of fit *S* are based on *F*^2^, conventional *R*-factors *R* are based on *F*, with *F* set to zero for negative *F*^2^. The threshold expression of *F*^2^ > σ(*F*^2^) is used only for calculating *R*-factors(gt) *etc*. and is not relevant to the choice of reflections for refinement. *R*-factors based on *F*^2^ are statistically about twice as large as those based on *F*, and *R*- factors based on ALL data will be even larger.

### Fractional atomic coordinates and isotropic or equivalent isotropic displacement parameters (Å^2^)

**Table:**

	*x*	*y*	*z*	*U*_iso_*/*U*_eq_	
S1	0.58190 (4)	0.11508 (6)	0.11107 (3)	0.04605 (16)	
N2	0.41409 (13)	0.10939 (19)	0.27382 (9)	0.0449 (3)	
N1	0.77113 (14)	0.1231 (2)	−0.00532 (10)	0.0532 (4)	
C1	0.73710 (15)	0.1181 (2)	0.08813 (11)	0.0453 (4)	
C4	0.62428 (15)	0.1069 (2)	0.22648 (11)	0.0460 (4)	
C6	0.32806 (15)	0.1149 (2)	0.34380 (11)	0.0438 (4)	
O2	0.68543 (14)	0.1159 (2)	−0.06611 (9)	0.0733 (4)	
C5	0.53109 (16)	0.1040 (3)	0.29481 (12)	0.0496 (4)	
C7	0.35846 (18)	0.1360 (3)	0.43745 (12)	0.0512 (4)	
C3	0.75216 (17)	0.1053 (3)	0.24363 (13)	0.0625 (5)	
O1	0.88152 (13)	0.1329 (3)	−0.02073 (10)	0.0807 (5)	
C2	0.81816 (17)	0.1119 (3)	0.16344 (13)	0.0581 (5)	
O3	0.16507 (15)	0.0835 (3)	0.22346 (11)	0.0904 (6)	
C8	0.26579 (19)	0.1470 (3)	0.49960 (13)	0.0574 (5)	
C11	0.20103 (17)	0.1045 (3)	0.31379 (13)	0.0584 (5)	
C9	0.1415 (2)	0.1387 (3)	0.46888 (15)	0.0665 (5)	
C10	0.1095 (2)	0.1179 (4)	0.37699 (17)	0.0748 (6)	
H2	0.904 (2)	0.113 (3)	0.1586 (14)	0.068 (6)*	
H3	0.787 (2)	0.107 (3)	0.2986 (17)	0.073 (7)*	
H5	0.564 (2)	0.096 (3)	0.3560 (17)	0.079 (7)*	
H7	0.447 (2)	0.150 (3)	0.4606 (13)	0.063 (6)*	
H10	0.028 (3)	0.112 (4)	0.3543 (19)	0.100 (9)*	
H9	0.080 (2)	0.151 (3)	0.5109 (16)	0.078 (7)*	
H8	0.288 (2)	0.167 (3)	0.5609 (16)	0.069 (6)*	
H1	0.227 (3)	0.069 (4)	0.198 (2)	0.097 (10)*	

### Atomic displacement parameters (Å^2^)

**Table:**

	*U*^11^	*U*^22^	*U*^33^	*U*^12^	*U*^13^	*U*^23^
S1	0.0373 (2)	0.0531 (3)	0.0481 (2)	−0.00158 (18)	0.00516 (15)	0.00117 (19)
N2	0.0434 (7)	0.0456 (8)	0.0468 (7)	−0.0009 (6)	0.0106 (5)	−0.0022 (6)
N1	0.0513 (8)	0.0589 (9)	0.0506 (8)	−0.0077 (7)	0.0128 (6)	0.0009 (7)
C1	0.0413 (8)	0.0481 (9)	0.0475 (8)	−0.0043 (7)	0.0102 (6)	−0.0017 (7)
C4	0.0415 (8)	0.0509 (10)	0.0463 (8)	−0.0008 (7)	0.0083 (6)	−0.0016 (7)
C6	0.0416 (8)	0.0412 (8)	0.0496 (8)	0.0020 (7)	0.0113 (6)	0.0011 (7)
O2	0.0625 (8)	0.1093 (12)	0.0482 (7)	−0.0094 (8)	0.0035 (6)	0.0076 (7)
C5	0.0433 (9)	0.0598 (11)	0.0463 (9)	0.0000 (8)	0.0086 (7)	−0.0012 (8)
C7	0.0498 (9)	0.0535 (11)	0.0510 (9)	0.0034 (8)	0.0087 (7)	0.0005 (8)
C3	0.0435 (9)	0.0957 (16)	0.0482 (10)	−0.0015 (9)	0.0028 (7)	−0.0021 (10)
O1	0.0533 (8)	0.1232 (14)	0.0681 (9)	−0.0151 (8)	0.0234 (7)	−0.0032 (9)
C2	0.0368 (8)	0.0814 (13)	0.0567 (10)	−0.0030 (9)	0.0072 (7)	−0.0037 (9)
O3	0.0490 (8)	0.160 (2)	0.0621 (9)	0.0045 (10)	0.0003 (7)	−0.0177 (10)
C8	0.0652 (11)	0.0581 (12)	0.0506 (10)	0.0055 (9)	0.0174 (9)	0.0026 (8)
C11	0.0448 (9)	0.0739 (13)	0.0572 (10)	−0.0007 (9)	0.0077 (7)	−0.0046 (9)
C9	0.0600 (11)	0.0721 (14)	0.0707 (13)	0.0035 (10)	0.0295 (10)	0.0010 (10)
C10	0.0410 (10)	0.1066 (19)	0.0783 (14)	−0.0021 (11)	0.0147 (9)	−0.0058 (13)

### Geometric parameters (Å, º)

**Table:**

S1—C1	1.7054 (18)	C7—C8	1.380 (3)
S1—C4	1.7107 (18)	C7—H7	0.99 (2)
N2—C5	1.264 (2)	C3—C2	1.399 (3)
N2—C6	1.411 (2)	C3—H3	0.86 (2)
N1—O1	1.212 (2)	C2—H2	0.92 (2)
N1—O2	1.231 (2)	O3—C11	1.353 (2)
N1—C1	1.428 (2)	O3—H1	0.78 (3)
C1—C2	1.352 (3)	C8—C9	1.370 (3)
C4—C3	1.368 (3)	C8—H8	0.92 (2)
C4—C5	1.447 (2)	C11—C10	1.383 (3)
C6—C7	1.387 (3)	C9—C10	1.366 (3)
C6—C11	1.396 (2)	C9—H9	0.93 (2)
C5—H5	0.94 (2)	C10—H10	0.91 (3)
			
C1—S1—C4	89.58 (8)	C4—C3—C2	113.16 (17)
C5—N2—C6	120.03 (15)	C4—C3—H3	122.7 (16)
O1—N1—O2	123.60 (15)	C2—C3—H3	124.1 (16)
O1—N1—C1	118.92 (15)	C1—C2—C3	110.35 (16)
O2—N1—C1	117.48 (15)	C1—C2—H2	121.6 (13)
C2—C1—N1	125.76 (16)	C3—C2—H2	128.0 (13)
C2—C1—S1	114.73 (13)	C11—O3—H1	106 (2)
N1—C1—S1	119.50 (13)	C9—C8—C7	119.92 (19)
C3—C4—C5	126.26 (16)	C9—C8—H8	120.7 (14)
C3—C4—S1	112.18 (13)	C7—C8—H8	119.3 (14)
C5—C4—S1	121.56 (13)	O3—C11—C10	118.94 (18)
C7—C6—C11	118.31 (16)	O3—C11—C6	121.26 (17)
C7—C6—N2	126.03 (16)	C10—C11—C6	119.80 (18)
C11—C6—N2	115.62 (15)	C10—C9—C8	120.01 (18)
N2—C5—C4	122.75 (16)	C10—C9—H9	120.7 (14)
N2—C5—H5	122.4 (15)	C8—C9—H9	119.3 (14)
C4—C5—H5	114.9 (15)	C9—C10—C11	120.9 (2)
C8—C7—C6	121.03 (18)	C9—C10—H10	122.2 (18)
C8—C7—H7	118.6 (12)	C11—C10—H10	116.8 (18)
C6—C7—H7	120.3 (12)		
			
O1—N1—C1—C2	−4.2 (3)	C5—C4—C3—C2	−179.07 (19)
O2—N1—C1—C2	175.3 (2)	S1—C4—C3—C2	0.4 (2)
O1—N1—C1—S1	177.06 (14)	N1—C1—C2—C3	−179.06 (17)
O2—N1—C1—S1	−3.4 (2)	S1—C1—C2—C3	−0.3 (2)
C4—S1—C1—C2	0.44 (16)	C4—C3—C2—C1	0.0 (3)
C4—S1—C1—N1	179.27 (14)	C6—C7—C8—C9	−0.6 (3)
C1—S1—C4—C3	−0.44 (16)	C7—C6—C11—O3	−179.9 (2)
C1—S1—C4—C5	179.02 (15)	N2—C6—C11—O3	2.3 (3)
C5—N2—C6—C7	7.5 (3)	C7—C6—C11—C10	0.9 (3)
C5—N2—C6—C11	−174.84 (17)	N2—C6—C11—C10	−176.96 (19)
C6—N2—C5—C4	−177.11 (16)	C7—C8—C9—C10	0.6 (3)
C3—C4—C5—N2	178.60 (19)	C8—C9—C10—C11	0.1 (4)
S1—C4—C5—N2	−0.8 (3)	O3—C11—C10—C9	179.8 (2)
C11—C6—C7—C8	−0.1 (3)	C6—C11—C10—C9	−0.9 (4)
N2—C6—C7—C8	177.44 (17)		

### Hydrogen-bond geometry (Å, º)

**Table:**

*D*—H···*A*	*D*—H	H···*A*	*D*···*A*	*D*—H···*A*
O3—H1···O2^i^	0.78 (3)	2.54 (3)	3.192 (3)	141 (3)
O3—H1···N2	0.78 (3)	2.23 (3)	2.711 (2)	121 (3)

Symmetry code: (i) −*x*+1, −*y*, −*z*.
