# Supplementary figures and images for: Crystal structure of 2-{[(5-nitro­thio­phen-2-yl)methyl­idene]amino}­phenol
Source: Acta Crystallogr E Crystallogr Commun. 2015 May 23;71(Pt 6):o418. doi: 10.1107/S2056989015009202 (PMC4459297; doi:10.1107/S2056989015009202)

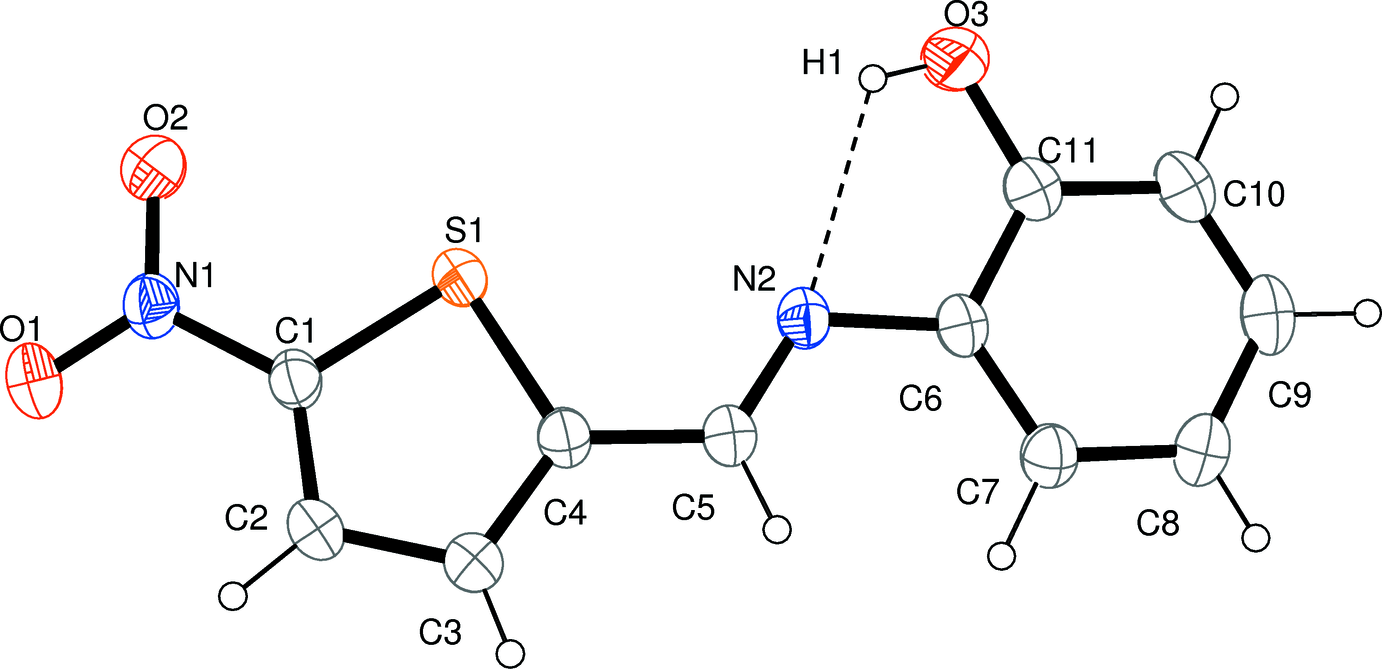

Supplement: Supplementary file 4 [file e-71-0o418-fig1.tif]

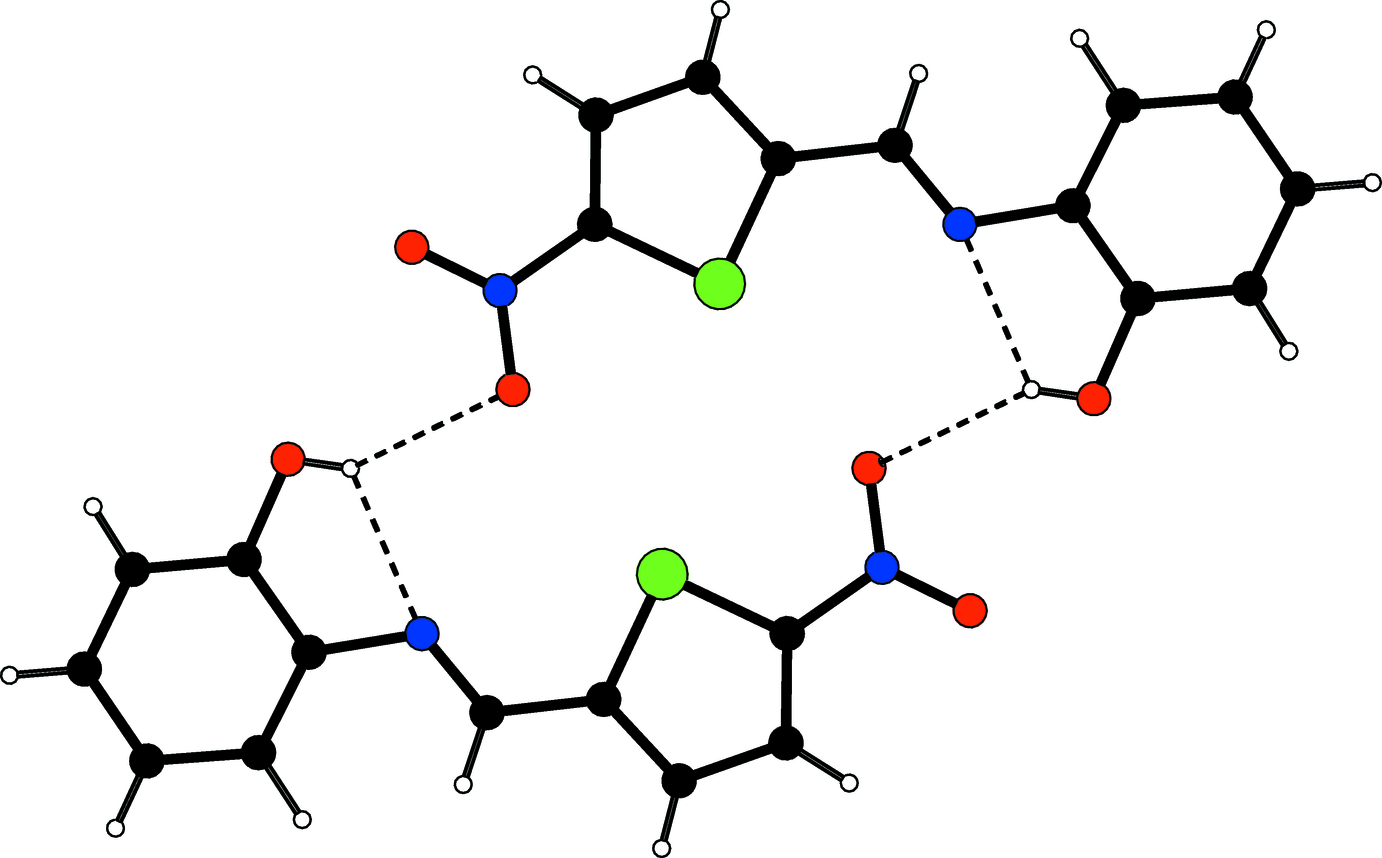

Supplement: Supplementary file 5 [file e-71-0o418-fig2.tif]

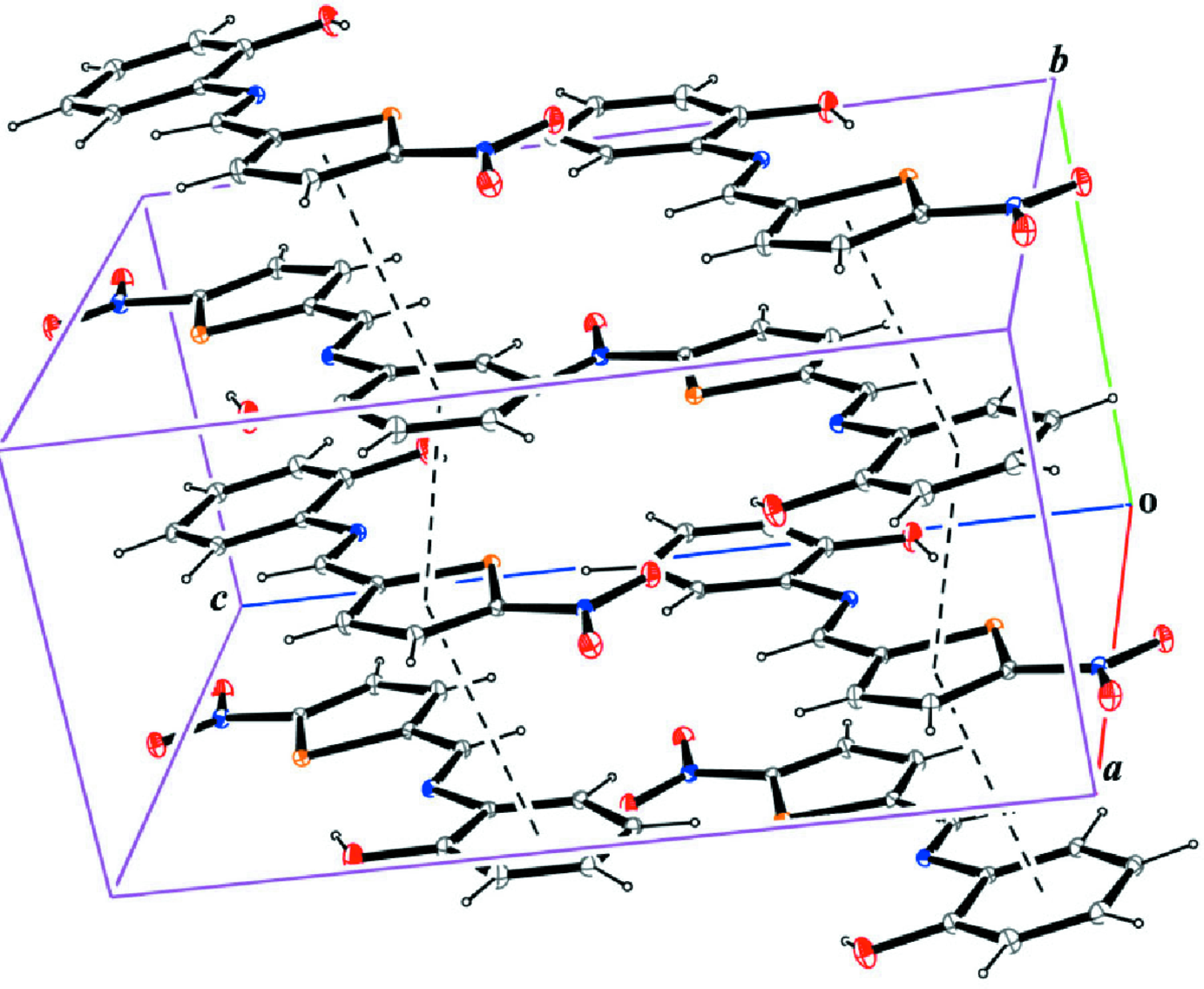

Supplement: Supplementary file 6 [file e-71-0o418-fig3.tif]
